# Longitudinal Measurement Properties of the Montreal Cognitive Assessment

**DOI:** 10.1093/geroni/igab046.047

**Published:** 2021-12-17

**Authors:** Björn Andersson, Hao Luo, Gloria H Y Wong, Terry Y S Lum

**Affiliations:** 1 University of Oslo, Oslo, Oslo, Norway; 2 The University of Hong Kong, Hong Kong, Hong Kong

## Abstract

Background: The Montreal Cognitive Assessment (MoCA) has started to be widely used in longitudinal investigations to measure changes in cognition. However, the longitudinal measurement properties of MoCA have not been investigated. We aimed to examine the measurement invariance of individual MoCA items across four time points. Methods: We used longitudinal data collected between 2014 and 2017 from a cohort study on health and well-being of older adults in Hong Kong. The Cantonese version of the MoCA was used. We applied multiple group confirmatory factor analysis of ordinal variables to examine measurement invariance by educational level and across time points. Invariant items were identified by sequential model comparisons. Results: We included 1029 participants that answered MoCA items across all time points. We found that items Cube, Clock Hand and Clock Number had significantly different item parameters between participants with and without formal education at all time points. The selected model (RMSEA=0.031; SRMR=0.064) indicated that eight items (Trail, Cube, Clock Shape, Clock Number, Clock Hand, Abstraction, Short-term Memory, and Orientation) did not exhibit measurement invariance over time. However, the differences in item parameter estimate over time were marginal. Accounting for the lack of measurement invariance did not substantially affect classification properties based on cutoff values at the 2nd ( major neurocognitive disorder) and 7th (mild cognitive impairment) percentile. Conclusion: Our findings support using MoCA to assess changes in cognition over time in the study population. Future research should examine the longitudinal measurement properties of the test in other populations with different characteristics.

